# Brain Network Regional Synchrony Analysis in Deafness

**DOI:** 10.1155/2018/6547848

**Published:** 2018-04-29

**Authors:** Lei Xu, Chang-Dong Wang, Mao-Jin Liang, Yue-Xin Cai, Yi-Qing Zheng

**Affiliations:** ^1^School of Data and Computer Science, Sun Yat-sen University, Guangzhou, China; ^2^Department of Otolaryngology, Sun Yat-sen Memorial Hospital, Guangzhou, China; ^3^Institute of Hearing and Speech-Language Science, Sun Yat-sen University, Guangzhou, China

## Abstract

Deafness, the most common auditory disease, has greatly affected people for a long time. The major treatment for deafness is cochlear implantation (CI). However, till today, there is still a lack of objective and precise indicator serving as evaluation of the effectiveness of the cochlear implantation. The goal of this EEG-based study is to effectively distinguish CI children from those prelingual deafened children without cochlear implantation. The proposed method is based on the functional connectivity analysis, which focuses on the brain network regional synchrony. Specifically, we compute the functional connectivity between each channel pair first. Then, we quantify the brain network synchrony among regions of interests (ROIs), where both intraregional synchrony and interregional synchrony are computed. And finally the synchrony values are concatenated to form the feature vector for the SVM classifier. What is more, we develop a new ROI partition method of 128-channel EEG recording system. That is, both the existing ROI partition method and the proposed ROI partition method are used in the experiments. Compared with the existing EEG signal classification methods, our proposed method has achieved significant improvements as large as 87.20% and 86.30% when the existing ROI partition method and the proposed ROI partition method are used, respectively. It further demonstrates that the new ROI partition method is comparable to the existing ROI partition method.

## 1. Introduction

Deafness, also known as hearing impairment, is always one of the most common auditory diseases. Reported in a survey, as of 2013, deafness influences about 1.1 billion people to a certain extent [1]. Deafness greatly plagues people, because it not only causes work-related obstacles but also results in mental illness [2]. To make matters worse, for some children who are prelingually deafened, hearing loss can make them lose ability to learn spoken language. Fortunately, cochlear implantation is the effective way to treat congenital severe deafness [3]. However, whether the cochlear implantation is successful mostly depends on children's own subjective feeling and, what is worse, for children of very small age, they are not able to express their feelings clearly. Up to now, there is still a lack of effective and objective approaches for the evaluation of the effectiveness of the cochlear implantation. Recently, with the rapid growth of noninvasive techniques such as electroencephalogram (EEG) [4–6], magnetoencephalogram (MEG) [7, 8], and functional magnetic resonance imaging (fMRI) [9, 10], these recording techniques are regarded as useful tools in clinical neuroscience. Compared to other techniques, EEG is more widely used for the auditory diseases because of its simplicity, low cost, and high temporal resolution [11, 12]. An event-related potential (ERP) report has demonstrated that deaf individuals will have a larger ERP amplitude and an earlier distribution of brain activity when they are engaged in visual stimuli [13]. Besides, some efforts have been made to investigate the brain activity of children who have worn cochlear implant by studying ERP. For example, in [14], higher P2 amplitude over visual occipital cortices during the visual task explains that the brain enhances the activity within Visual Cortex to make up for deficient auditory stimuli provided by the CI. Similarly, in another experiment [15], the cross modal plasticity in deafness is also confirmed through observing the P1 and N1 components of the cortical auditory evoked potential (CAEP) in CI children. In addition, the findings of [6] demonstrate that P2 latency of the visual evoked potentials (VEPs) develops with CI usage, and may be a biomarker of Visual Cortex plasticity. Nevertheless, the previous studies emphasize more on the EEG signal features, for example, the amplitude and latency of peak value, without taking the brain connectivity or the network perspective into account. Because these studies have reported that cortical plasticity occurred in prelingual profound hearing subjects and subjects who received CI, so we want to investigate the brain network connectivity among cortex regions.

The main contributions of this paper can be summarized as follows:

1. We propose a method called ROISmining (ROI Synchrony mining) by computing the EEG synchrony among ROIs from well-preprocessed EEG signals, where both intraregional synchrony and interregional synchrony are computed.
2. We design a new ROI partition method and the experiment results show that our proposed partition method is comparable to the existing ROI partition method.
3. Compared to other methods, our proposed method achieves a higher accuracy, which can better differentiate cochlear implant children from controls.

The rest of the paper is organized as follows. In Section 2, we make a survey of the related work.  Section 3 introduces the EEG dataset and the details of the proposed ROISmining method, including computing EEG synchrony among channels, quantifying EEG regional synchrony, and classification. Experimental results are reported and discussed in Section 4. Finally, we draw a conclusion in Section 5.

## 2. Related Work

In the literature, many methods for EEG signal feature extraction have been proposed and used in the clinical applications such as epileptic seizure classification, detection of Alzheimer's disease, and auxiliary therapy for tinnitus [16–20]. In [16], a method of automatic analysis of EEG signals, which combines wavelet transform, principal component analysis (PCA), and support vector machine (SVM), was proposed to classify states of seizure. A method for EEG signal processing named Empirical Mode Decomposition (EMD) was developed for analyzing nonlinear and nonstationary EEG data [17, 18]. Besides, entropy is also a significant nonlinear parameter that reflects the complexity of the EEG signal, so the review [19] presents the application of various entropies for diagnosis of epilepsy. Recently, it becomes increasingly popular to combine the characteristic features from different methods to make a more accurate prediction, as reviewed by Giannakakis et al. [20]. However, all the aforementioned methods focus more on mining the discriminative characteristics of EEG signal, without considering the connectivity between EEG signal.

The functions of brain mostly rely on the capability of neurons to transmit electrochemical information to other cells and their capability to react to the information received from other cells. In other words, the output of brain results from collaboration or inhibition of different brain areas. Therefore, unlike the EEG feature extraction methods, some methods pay more attention to the pairwise connectivities of EEG channels or the brain network mining [21–23]. In [21], bivariate features of EEG synchronization from all channel pairs were trained to discriminate seizure state. Liu et al. [22] calculated functional connectivity to construct brain network and the results showed that brain networks of normal control subjects possessed small-world topological properties whereas these properties were changed in attention-deficit/hyperactivity disorder (ADHD). Cao et al. [23] proposed a semisupervised brain network analysis approach based on constrained tensor factorization. However, the aforementioned methods only consider the pairwise connectivities of EEG channels, which neglect the regional connectivities. Compared with pairwise connectivities of channels, regional connectivities can be considered as large-scale synchrony [24], which contains more information from the regional function perspective. In this study, we propose a new method called ROISmining (ROI Synchrony mining), which for the first time utilizes the brain network regional synchrony to differentiate cochlear implanted children from controls and is shown to perform better for clinical treatment of deafness.

## 3. Methods

### 3.1. Methods Overview

Brain connectivity can be classified into effective connectivity (causal interactions), anatomical connectivity (anatomic links), and functional connectivity (statistical dependencies) [25]. Effective connectivity is defined as the direct or indirect effect that one neural system exerts over another [26]. Anatomical connectivity, also called structural connectivity, looks for physical connections in the brain. Unlike anatomical connectivity, the functional connectivity is highly time-dependent, which can be defined as the temporal correlation among neuronal signals.

In the brain, neurons in the same functional area of the brain are often connected to each other, and different functional areas also have connection between each other. Therefore, the connections between brain, which we also call “Cortico-cortical connections”, can be roughly classified in two groups: local connections linking neurons in the same cortical area and long-range connections between neurons of different cortical regions [27]. In general, functional connectivity is usually calculated between all channels, so the correlation matrices contain the local connectivity and long-range connectivity at the same time. However, both the local and long-range connectivity emphasize the link between two specific channels. In this paper, we will focus more on the regional connectivity, whose emphasis is the connectivity among regions, rather than the connectivity between a channel of a region and a channel of another region. Therefore, we develop a brain connectivity analysis method called ROISmining which investigates the functional connectivity between different functional regions. What is more, compared with synchronization of all channel pairs, synchronization of all region pairs is more reasonable, because it can not only provide complementary local information but also characterize the global information of the whole brain network.

**Figure 1:**
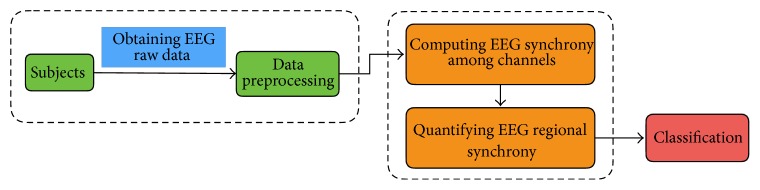
The overall framework of ROISmining.

The overall framework of the proposed ROISmining (ROI Synchrony mining) method is shown in Figure 1, which is composed of four main steps: (1) preprocessing; (2) computing EEG synchrony among channels; (3) quantifying EEG regional synchrony; (4) classification.

### 3.2. Dataset

25 subjects participated in the experiments including 17 cochlear implanted children (aged 5.08 years; standard deviation is 1.65 years) and 8 children (aged 6.23 years, standard deviation is 1.37 years) with congenital moderate-severe conductive hearing loss as the control group. All of participants are recruited from Sun Yat-sen Memorial Hospital. All the cochlear implanted children are implanted unilaterally for less than one year and at least 2 years old. None of the CI children with prelingual profound hearing loss participating in this study have a previous history of special infection, kernicterus, or ototoxic drug application or have inner ear or auditory malformation during preoperative CT and MRI evaluations. All the subjects are right-handed and have no visual problems. It is noteworthy that the written consent is obtained from the parents of all the subjects before the experiments. The demographic information of the 17 CI children is showed in Table 1.

In the present study, the dataset is visual evoked potentials (VEPs) of subjects. The reason for selecting visual stimuli experiment is that those non-CI children cannot receive and respond to the auditory stimulus. In recording, the visual stimuli are one photo with imaginative sound and one photo without imaginative sound. In addition, one atypical photo (a wolf picture) is presented to keep the subjects focused on the stimuli. Furthermore, to make sure the data has low noise, all subjects are asked to sit on a comfortable chair in front of a high-resolution VGA computer monitor, viewing distance of about 1 meter in a sound insulation and electromagnetic shielding room.

**Figure 2:**
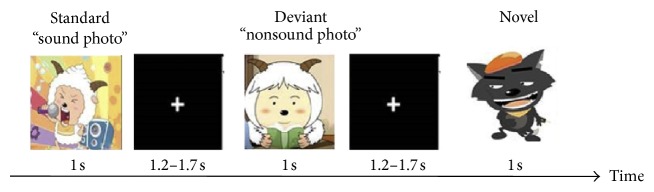
The experimental block of visual stimuli.

The experimental block is shown in Figure 2, which consists of an intermittent stimulus mode with 100 “sound photo” trials and 100 “nonsound photo” trials divided into two blocks. Each picture lasts for 1 second and is followed by blank screen, whose duration ranges from 1.2 seconds to 1.7 seconds. The subjects are instructed to concentrate their eyes on the picture and respond to the wolf picture by pressing the blank button of a handle. The sampling rate for the EEG recording is 1 kHz, and the impedances of electrodes are limited below 50 kΩ.

### 3.3. Preprocessing

The collected EEG signals are firstly bandpass filtered between 0.3 and 30 Hz and then segmented with 100 ms prestimulus and 600 ms poststimulus time, so the duration of epoch in each subject is 700 ms. If the amplitude of any electrode exceeds 75 *μ*V, the segment will be considered to have artifacts and then be rejected. Besides, the epochs which have any eye blinking (eye channel exceeded 140 *μ*V) or eye movement (eye channel exceeded 55 *μ*V) are also removed. After being rereferenced, bad channels are checked and removed from the recording. It is noteworthy that the EEG montage used in this study is all electrodes average reference. After the bad channels removal and artifact rejection, about 80–90 trails per subject for each “sound” and “nonsound” stimuli are included into subsequent analysis. Finally, the “sound photo” and “nonsound photo” stimuli are added together.

### 3.4. Computing EEG Synchrony among Channels

One of the most significant characteristics of the brain is not the number of neurons that it contains, but the connectivity between these neurons. In order to calculate the synchronization of EEG recordings, a number of measures are utilized in the scientific literature. What needs to be mentioned here is that the connectivity to be computed is the functional connectivity, so it is not necessary to consider the additional causal information. At present, there exist many measures to calculate the functional connectivity and we have also tried a lot in our experiments. However, considering stationarity, we choose four measures which perform best in our experiments. They are Pearson's correlation coefficient, cross-correlation function, magnitude squared coherence, and phase locking value. What is more, the measures mentioned above are more commonly used measure in the literature [28–31].

It should be pointed out that we first obtain the averaged waveform from all trials and then calculate synchronization measures over the averaged waveform for each subject.

#### 3.4.1. Pearson Correlation Coefficient (COR)

Pearson correlation coefficient is a simple and effective method to depict the linear correlation between two time series. For any two time series *x* and *y*, the correlation coefficient is computed as follows [28]:

(1) $$\begin{array}{c} r=\frac{1}{N}\overset{N}{\underset{n=1}{\sum }}\frac{\left(x\left(n\right)-\bar{x}\right)}{\sigma_{x}}\frac{\left(y\left(n\right)-\bar{y}\right)}{\sigma_{y}}, \end{array}$$

where *N* is the length of the signals, $\bar{x}$ and $\bar{y}$ are the mean values of the time series *x* and *y*, and *σ*_*x*_ and *σ*_*y*_ represent the standard deviation of the time series *x* and *y*.

The range of *r* is as follows: −1 ≤ *r* ≤ 1. If the two time series are correlated to each other closely, then the correlation coefficient between these two signals is positive and close to 1. In contrast, if there exists inverse linear correlation between these two time series, the correlation coefficient is near −1. If there does not exist linear interdependence of the two time series, *r* = 0.

#### 3.4.2. Cross-Correlation Coefficient (XCOR)

Cross-correlation coefficient is also a measure of linear correlation of two time series *x* and *y*. But, different from Pearson correlation, cross correlation is calculated as a function of one delayed signal relative to the other signal. The cross-correlation coefficient is computed as follows [29]:

(2) $$\begin{array}{c} r\left(\;\tau \;\right)=\frac{1}{N-\tau }\overset{N-\tau }{\underset{n=1}{\sum }}\frac{\left(x\left(n+\tau \right)-\bar{x}\right)}{\sigma_{x}}\frac{\left(y\left(n\right)-\bar{y}\right)}{\sigma_{y}}, \end{array}$$

where *τ* is the time delay and the definitions of *N*, $\bar{x}$, $\bar{y}$, *σ*_*x*_, and *σ*_*y*_ are the same as (1).

#### 3.4.3. Magnitude Squared Coherence (COH)

Magnitude squared coherence is used to estimate the relation between two time series in frequency domain. In order to compute coherence, we compute the cross-spectrum of the signals first. The cross-spectrum *P*_*xy*_(*f*) between the two signals *x* and *y* is defined as follows [30]:

(3) $$\begin{array}{c} P_{xy}\left(\;f\;\right)=X\left(\;f\;\right)Y^{*}\left(\;f\;\right), \end{array}$$

where *X*(*f*) and *Y*(*f*) are the Discrete Fourier Transform (DFT) of the signals *x* and *y*, respectively, and *Y*^*∗*^(*f*) is the complex conjugate of *Y*(*f*) with *f* being the frequency interval.

Using the cross-spectrum, the coherence is a real-valued function expressed as follows:

(4) $$\begin{array}{c} c\left(\;f\;\right)=\frac{{\left|P_{xy}\left(f\right)\right|}^{2}}{P_{xx}\left(f\right)P_{yy}\left(f\right)} \end{array}$$

which takes value from 0 to 1 (i.e., *c*(*f*) ∈ [0,1]), where 1 means that there exists high synchronization between two time signals while 0 indicates no relation between them.

The advantages of the aforementioned measures are simplicity and low complexity, yet they only examine linear relation. Therefore, we introduce a measure which evaluates the synchronization between two time series by using the instantaneous phase of the signals.

#### 3.4.4. Phase Locking Value (PLV)

Phase locking value only measures the phase value, which fully utilizes the phase difference between signals, even when the amplitudes of signals are statistically independent. The key problem of computing PLV is to calculate the instantaneous phase of the signal. According to [31], the instantaneous phase *ϕ*_*x*_ of the signal *x* is defined as follows:

(5) $$\begin{array}{c} \phi_{x}\left(\;n\;\right)=\arg\left[\;x\left(\;n\;\right)+i\tilde{x}\left(\;n\;\right)\;\right], \end{array}$$

where $\tilde{x}$ denotes the Hilbert Transform of *x*, arg⁡[*∗*] denotes the function of computing the angular component of a complex number, and *i* denotes the imaginary unit.

Then, we can use the relative instantaneous phase difference of signals *x* and *y* to compute the PLV index, which is defined as follows [32]:

(6) $$\begin{array}{c} \gamma =\left|\;\frac{1}{N}\overset{N}{\underset{n=1}{\sum }}e^{i\left(\phi_{x}\left(n\right)-\phi_{y}\left(n\right)\right)}\;\right|. \end{array}$$

In experiments, all the aforementioned measures are used to test the performance. In addition, the parameter analysis experiments are also conducted to analyze the effect of the parameter time delay *τ* on XCOR measure and the parameter frequency interval *f* on COH measure.

It is noteworthy that the measures mentioned above are used separately to extract the synchronization feature.

### 3.5. Quantifying EEG Regional Synchrony

The main idea of our proposed method is to obtain EEG regional synchrony of different ROIs and then aggregate them as features for classification. Because some previous studies [6, 14, 15] have reported that cortical plasticity occurred in both of the prelingual profound hearing subjects and the subjects who received CI, therefore, all the cortex regions should be taken into account in the analysis. Thus, it is necessary to introduce the ROI partition method first.

#### 3.5.1. The ROI Partition Methods

**Figure 3:**
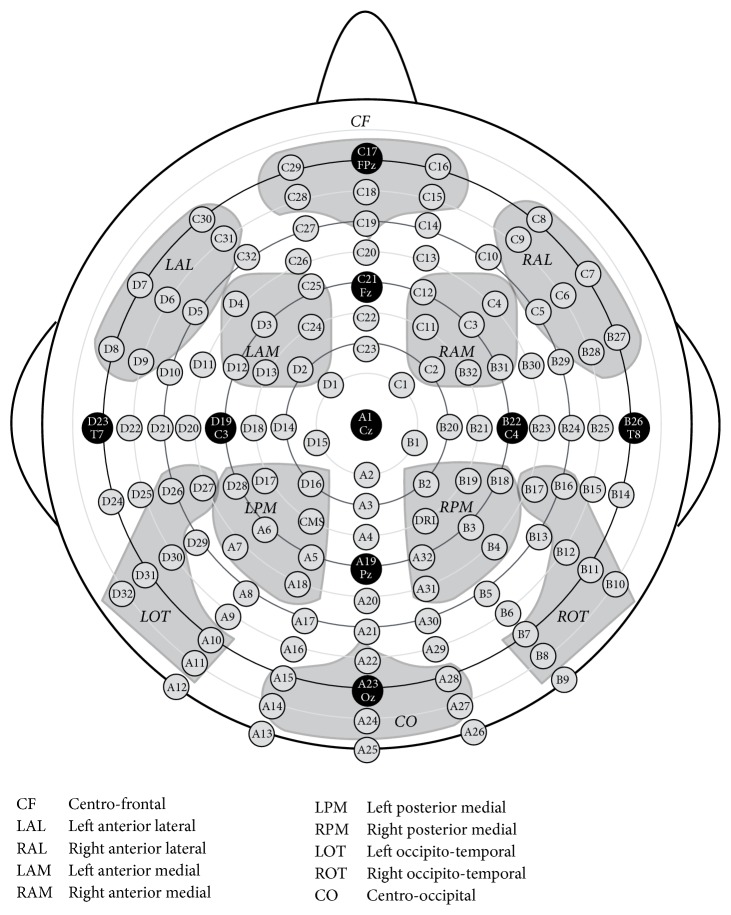
ROI partition method I: the locations of 128 electrode sites and the 10 designated regions of interest (ROIs).

First of all, we will describe the ROI partition method introduced in [33], where 10 regions of interest across the scalp of 128-channel EEG recording system were designated and labeled as Centrofrontal (CF), Left Anterior Lateral (LAL), Right Anterior Lateral (RAL), Left Anterior Medial (LAM), Right Anterior Medial (RAM), Left Posterior Medial (LPM), Right Posterior Medial (RPM), Left Occipitotemporal (LOT), Right Occipitotemporal (ROT), and Centrooccipital (CO). The locations of 128 electrode points and the corresponding 10 ROIs are shown in Figure 3. Each region contains 7 electrode sites, and therefore 70 of 128 channels will be selected.

**Figure 4:**
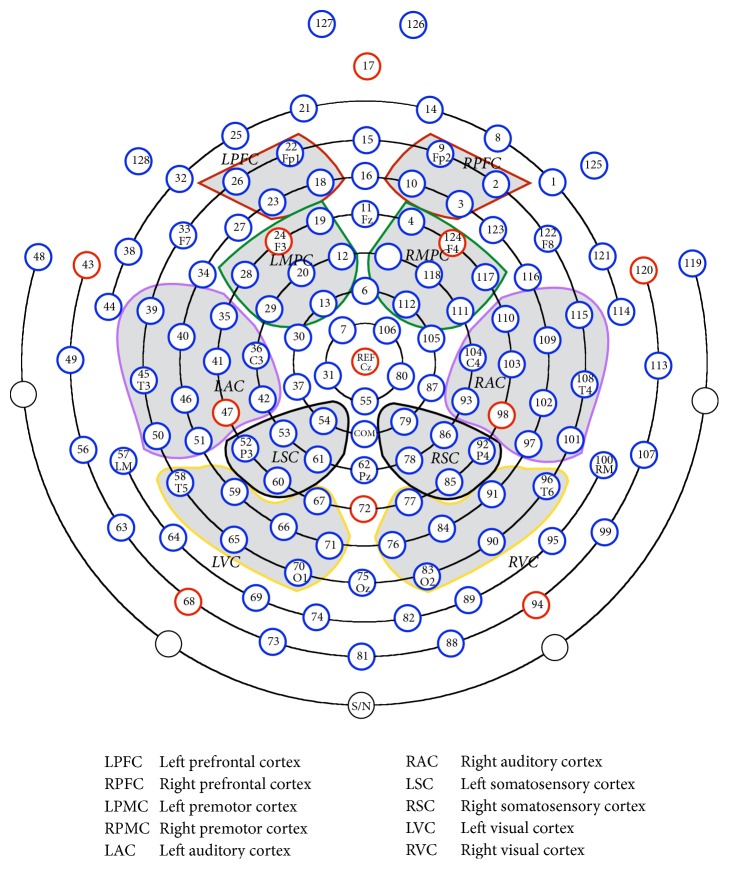
ROI partition method II: The locations of 128 electrode sites and the 10 designated regions of interest (ROIs).

Apart from the above partition method, we propose a new partition method which is also based on the location of functional brain regions and will be shown to be more suitable for analysis of auditory disease. Similarly, the 128-channel EEG recording system is divided into 10 regions across the scalp. The ten regions are also symmetric as shown in Figure 4.

1. PFC (Prefrontal Cortex) is primarily responsible for brain functions, including thinking, perception, information memory, and attention.
2. PMC (Premotor Cortex) is an area of motor cortex lying within the frontal lobe of the brain and it mainly plays the role of controlling eye movement.
3. AC (Auditory Cortex) is in charge of processing auditory information in the brain.
4. VC (Visual Cortex) is in charge of processing visual information in the brain.
5. SC (Somatosensory Cortex) is mainly related to receiving tactile stimuli.

If the new partition method is applied, then 68 of 128 channels will be chosen.

#### 3.5.2. Intraregional and Interregional Synchrony

Because the synchronization measures between the selected signal pairs have been computed as introduced in Section 3.4, then we can quantify the EEG regional synchrony of the 10 designated ROIs pairs. However, when we regard a region as a cluster of several channels, the synchronization measure between the channels in the same region should also be considered. Based on the above considerations, our proposed method evaluates the EEG regional synchrony from the following two aspects.

(*1) Intraregional Synchrony*. Because each region contains several electrode points (channels), we first evaluate the intraregional synchrony of each region. As neurons in the same region have the same function, based on the idea of isomorphism, we compute the average value of the functional connectivity among each channel pair which is located in the same region as the intraregional synchrony of this region. Therefore, this process can be expressed as

(7) $$\begin{array}{c} Sync_{R_{k}}=\frac{\sum_{i=1}^{N_{k}}\sum_{j=i+1}^{N_{k}}Sync_{e_{i},e_{j}}}{N_{k}*\left(N_{k}-1\right)/2}, \end{array}$$

where *N*_*k*_ is the number of channels in region *k*, *e*_*i*_ and *e*_*j*_ are the different channels of the region *k*, and Sync_*e*_*i*_,*e*_*j*__ denotes the synchronization measure between channels *e*_*i*_ and *e*_*j*_. 

(*2) Interregional Synchrony*. Second, we evaluate the interregional synchrony between two different regions. From the micro perspective, since the connectivity between two different regions is actually the communication among neurons in different regions, we define the interregional synchrony as the average value of the synchronization measure between each channel pair across the two different regions. In addition, this approach can compensate for the loss of anatomical connection to some extent. The computational process is defined as

(8) $$\begin{array}{c} Sync_{R_{k},R_{l}}=\frac{\sum_{i=1}^{N_{k}}\sum_{j=1}^{N_{l}}Sync_{e_{i},e_{j}}}{N_{k}*N_{j}}, \end{array}$$

where *N*_*k*_ and *N*_*l*_ are the number of channels of region *k* and region *l*, respectively, and channel *e*_*i*_ is from region *k* while channel *e*_*j*_ is from region *l*.

As the number of regions is 10, in total 10 intraregional synchronies and 10*∗*9/2 = 45 interregional synchronies are quantified. Then these 55 EEG regional synchronies will be concatenated to form the discriminative feature vector of each subject.

### 3.6. Classifier

#### 3.6.1. *k*-Nearest Neighbors (*k*NN)

KNN is a lazy learning algorithm and it is also one of the simplest machine learning algorithms. Its main idea is that the label of an object is decided by a majority vote of its neighbors, meaning that the object is assigned to the most common category among its *k* nearest neighbors. KNN is simple, easy to understand, and easy to implement. And there is no need to estimate parameters and there is no training process. The key problem to the algorithm is the choice of *k*, which depends upon the data. Usually, it is advisable to choose *k* to be an odd number rather than an even number to avoid trivial votes and make the results more reasonable. Considering that the number of subjects is relatively small, the optimal value of *k* is set as 1 by trial and error.

#### 3.6.2. Support Vector Machine (SVM) 

In the field of machine learning, SVM is a classical supervised model for pattern recognition and classification [34]. The main idea of SVM is implicitly mapping the original sample space into a high-dimensional space through kernel function, so the nonlinearly separable problem in the original space can be changed into a linearly separable problem in the new space. The final decision function of SVM is only determined by a few support vectors. Therefore, its computational complexity depends on the number of support vectors rather than the dimensions of the input, which makes SVM perform well, even when the given samples are not enough. So, we choose support vector machine as the classifier in our experiment for the reason that the number of subjects is relatively small.

### 3.7. Performance Evaluation

The performance of classification is evaluated by four evaluation measures, namely, accuracy, recall, precision, and *F*1 [35].

(9) Accuracy=TP+TNTP+TN+FP+FNRecall=TPTP+FNPrecision=TPTP+FPF1=2∗Precision∗RecallPrecision+Recall,

where TP (True Positive) is the number of positive samples which are correctly classified; TN (True Negative) is the number of negative samples which are correctly classified; FP (False Positive) is the number of positive samples which are misjudged; and FN (False Negative) is the number of negative samples which are misjudged. In the present experiment, the prelingually deafened subjects after cochlear implantation are labeled as positive, while the prelingually deafened subjects without cochlear implantation are labeled as negative. The higher value of evaluation measures indicates better classification results.

## 4. Results and Discussion

In this section, extensive experiments will be conducted to evaluate the proposed method on the real-world dataset collected in our previous work [6]. The programming language we use is MATLAB and the toolbox used for the computation of the synchronization measures is HERMES [29]. Both of the parameter analysis and comparison with other methods will be reported.

### 4.1. Parameter Analysis

**Figure 5:**
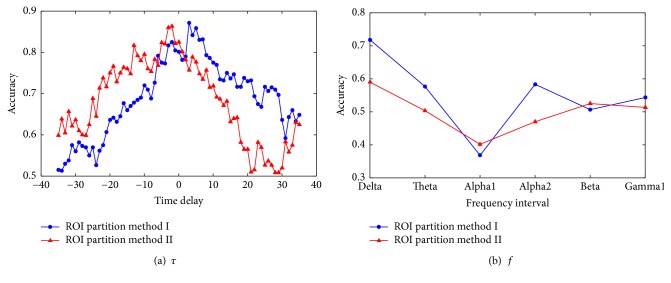
Parameter analysis: the effect of *τ*, the parameter in calculating XCOR, and *f*, the frequency interval in computing COH when using KNN algorithm.

**Figure 6:**
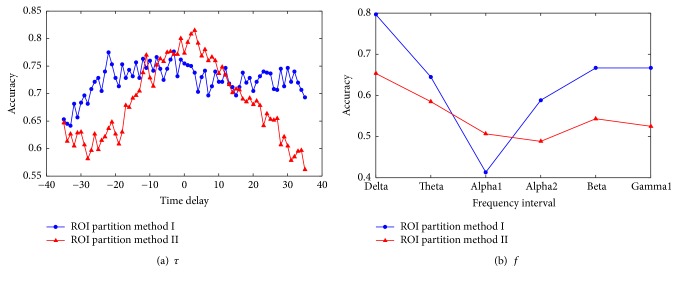
Parameter analysis: the effect of *τ*, the parameter in calculating XCOR, and *f*, the frequency interval in computing COH when using SVM algorithm.

In this subsection, parameter analysis will be conducted to show the effect of *τ*, the time delay in calculating XCOR value, and *f*, the frequency interval in computing COH value. For the two ROI partition methods, the influence on accuracy is shown in Figures 5 and 6, where the time delay *τ* varies from −35 to 35. Because EEG signals have been bandpass filtered between 0.3 and 30 Hz at the preprocessing stage, only six different frequency bands are used to compute COH, namely, Delta (1–4 Hz), Theta (4–8 Hz), Alpha 1 (8–10 Hz), Alpha 2 (10–13 Hz), Beta (14–25 Hz), and Gamma 1 (25–45 Hz).  Figure 5 shows the result of the KNN classifier with *k* = 1 while Figure 6 shows the result of the SVM classifier. Blue lines denote the result using the ROI partition method I and red lines indicate the result using the ROI partition method II. The curve clearly indicates that when the time delay is between −10 and 10, the accuracy is relatively higher, while when the absolute value of time delay is larger than 10, the accuracy will decrease gradually. This is because the EEG signals of each channel are recorded at the same time. Therefore, when the time delay is high, the linear correlation between two EEG signals is small. On the other hand, the COH value is the highest when the frequency is limited in Delta band for both of the two ROI partition methods. It means that the recording EEG signal contains more low-frequency components, so the signal information in the low-frequency domain is more abundant.

### 4.2. Comparison Results

Since there are only a few EEG classification algorithms associated with auditory disease, two suitable EEG signal classification methods are used as baselines in this experiment, which are MainPhase + SVM and DWT + PCA + SVM.

- MainPhase: a method which extracts main phase of signals as features for the SVM classifier [5]
- DWT + PCA + SVM: a versatile signal processing and analysis framework for EEG. Within this framework, discrete wavelet transform (DWT) is used to decompose the signals into the frequency subbands and then principal components analysis (PCA) is used to reduce the dimension of data [36].

For all the methods, the best parameters are obtained by trial and error.

In the comparison experiment, to make sure that the test set is balanced, we randomly choose three CI children and three controls as the test set, and the remaining 19 subjects comprise the training set. This hold-out process is repeated 100 times and then the mean values and standard deviations of the four evaluation measures are reported and compared.

The classification performance is summarized in Table 2. It can be intuitively seen that our proposed method is significantly superior to the other compared methods on the task of distinguishing CI children, no matter which ROI partition method is used. From Table 2, we can clearly see that the effect of the KNN classifier is better than the effect of the SVM classifier. In four synchronization measures, XCOR performs best, which indicates that the EEG signal contains more linear components. It also shows that there is a certain delay when different regions of the brain correlate with each other. When using ROI partition method I, the best classification result is obtained by using XCOR as the synchronization measure and 1NN as classifier, and the accuracy is as high as 0.872. On the other hand, when using ROI partition method II, the best performance is also obtained when using XCOR and 1NN, where the accuracy can achieve 0.863. It shows that the two partition methods are equally good which means that our proposed partition method is comparable to the existing partition algorithms. In addition, it is noteworthy that the recall value is relatively high, which means that the prelingually deafened subjects after cochlear implantation will be more accurately predicted.

## 5. Conclusion

In this paper, we aim to investigate whether the EEG-based functional connectivity analysis could be considered as a valuable measure in detecting successful cochlear implantation. Previous study by Liu et al. [6] has found that the average P2 latency was longer in CI group than non-CI group, which indicated cortex plasticity occurred after successful cochlear implantation. However, there is still a lack of measure in distinguishing the successful cochlear implantation using individual EEG data. To solve this problem, we have proposed* ROISmining* to perform brain network regional synchrony analysis for deafness. Specifically, compared with the existing EEG signal classification methods, our proposed method has positive results with achieving classification accuracy as high as 87.20% and 86.3% in distinguishing the subjects who received successful cochlear implantation from the non-CI prelingually profound hearing children, which was valuable in the evaluation of the effectiveness of the cochlear implantation. More importantly, we design a new ROI partition method which is comparable to the existing partition method. In future research, this approach will be extended to help distinguish other auditory diseases such as tinnitus and some diseases associated with brain dysfunction.

## Figures and Tables

**Table 1:** Demographic information of cochlear implantation (CI) children.

Subject code (gender)	Duration of CI experience (years)	Age at implantation (years)	Age now (years)
CI1 (F)	0	2.60	2.60
CI2 (M)	0	3.61	3.61
CI3 (F)	0	3.61	3.61
CI4 (F)	0	3.84	3.84
CI5 (M)	0	4.65	4.65
CI6 (F)	0	5.02	5.02
CI7 (M)	0	6.30	6.30
CI8 (F)	0	9.16	9.16
CI9 (F)	0.74	4.25	4.99
CI10 (F)	0.75	3.04	3.79
CI11 (M)	0.81	5.01	5.82
CI12 (F)	0.81	5.10	5.91
CI13 (F)	0.82	2.69	3.51
CI14 (M)	0.82	6.86	7.68
CI15 (F)	0.86	4.14	5.00
CI16 (F)	0.87	4.10	4.97
CI17 (M)	0.97	5.05	6.02

**Table 2:** Comparison of classification performance in terms of accuracy, recall, precision, and *F*1 on the dataset over 100 runs: mean values and variances (in parentheses).

	Methods	Accuracy	Recall	Precision	*F*1
ROI partition method I	ROISmining (COR) + KNN	0.800 (0.025)	0.900 (0.035)	0.777 (0.032)	0.819 (0.023)
	ROISmining (XCOR) + KNN	0.872 (0.019)	0.930 (0.021)	0.869 (0.027)	0.883 (0.015)
	ROISmining (COH) + KNN	0.718 (0.021)	0.917 (0.021)	0.675 (0.020)	0.769 (0.014)
	ROISmining (PLV) + KNN	0.458 (0.018)	0.767 (0.048)	0.471 (0.008)	0.578 (0.016)

ROI partition method I	ROISmining (COR) + SVM	0.763 (0.022)	0.923 (0.029)	0.724 (0.026)	0.798 (0.018)
	ROISmining (XCOR) + SVM	0.787 (0.022)	0.927 (0.026)	0.756 (0.027)	0.817 (0.018)
	ROISmining (COH) + SVM	0.797 (0.020)	0.947 (0.017)	0.754 (0.023)	0.828 (0.013)
	ROISmining (PLV) + SVM	0.607 (0.165)	0.810 (0.210)	0.589 (0.139)	0.669 (0.142)

ROI partition method II	ROISmining (COR) + KNN	0.822 (0.015)	0.870 (0.031)	0.813 (0.021)	0.826 (0.016)
	ROISmining (XCOR) + KNN	0.863 (0.020)	0.870 (0.040)	0.877 (0.026)	0.859 (0.025)
	ROISmining (COH) + KNN	0.590 (0.016)	0.873 (0.035)	0.572 (0.013)	0.678 (0.011)
	ROISmining (PLV) + KNN	0.383 (0.012)	0.753 (0.051)	0.423 (0.006)	0.539 (0.015)

ROI partition method II	ROISmining (COR) + SVM	0.805 (0.016)	0.850 (0.034)	0.805 (0.024)	0.810 (0.017)
	ROISmining (XCOR) + SVM	0.815 (0.019)	0.867 (0.042)	0.812 (0.026)	0.818 (0.022)
	ROISmining (COH) + SVM	0.653 (0.027)	0.873 (0.033)	0.624 (0.022)	0.717 (0.018)
	ROISmining (PLV) + SVM	0.388 (0.013)	0.733 (0.054)	0.424 (0.006)	0.534 (0.016)

Compared methods	MainPhase + SVM	0.477 (0.027)	0.760 (0.054)	0.483 (0.011)	0.583 (0.019)
	DWT + PCA + SVM	0.503 (0.017)	0.837 (0.037)	0.502 (0.007)	0.623 (0.0126)
